# The Optokinetic Reflex as a Tool for Quantitative Analyses of Nervous System Function in Mice: Application to Genetic and Drug-Induced Variation

**DOI:** 10.1371/journal.pone.0002055

**Published:** 2008-04-30

**Authors:** Hugh Cahill, Jeremy Nathans

**Affiliations:** 1 Department of Molecular Biology and Genetics, Johns Hopkins University School of Medicine, Baltimore, Maryland, United States of America; 2 Department of Neuroscience, Johns Hopkins University School of Medicine, Baltimore, Maryland, United States of America; 3 Department of Ophthalmology, Johns Hopkins University School of Medicine, Baltimore, Maryland, United States of America; 4 Howard Hughes Medical Institute, Johns Hopkins University School of Medicine, Baltimore, Maryland, United States of America; University of Maryland, United States of America

## Abstract

The optokinetic reflex (OKR), which serves to stabilize a moving image on the retina, is a behavioral response that has many favorable attributes as a test of CNS function. The OKR requires no training, assesses the function of diverse CNS circuits, can be induced repeatedly with minimal fatigue or adaptation, and produces an electronic record that is readily and objectively quantifiable.

We describe a new type of OKR test apparatus in which computer-controlled visual stimuli and streamlined data analysis facilitate a relatively high throughput behavioral assay. We used this apparatus, in conjunction with infrared imaging, to quantify basic OKR stimulus-response characteristics for C57BL/6J and 129/SvEv mouse strains and for genetically engineered lines lacking one or more photoreceptor systems or with an alteration in cone spectral sensitivity. A second generation (F2) cross shows that the characteristic difference in OKR frequency between C57BL/6J and 129/SvEv is inherited as a polygenic trait. Finally, we demonstrate the sensitivity and high temporal resolution of the OKR for quantitative analysis of CNS drug action.

These experiments show that the mouse OKR is well suited for neurologic testing in the context of drug discovery and large-scale phenotyping programs.

## Introduction

The rapid growth in the number and variety of behavioral studies of mice–in the contexts of forward genetic screens, targeted mutagenesis, or preclinical drug testing-has put a premium on developing methods for quantifying nervous system function in this species [1]–[4]. In humans, the classic neurologic examination relies on eliciting specific motor responses to assess not only the motor system itself but also sensory and cognitive processes upstream of the motor system [5]. In mice, simple motor tasks such as grip strength and facility on a rotorod are routinely used to monitor basic neuromuscular function, and in the latter case, also cerebellar and vestibular functions [6]. However, many behaviors, such as the amount and pattern of movement within a cage, show significant variability on repeated trials and/or between genetically identical mice and can only be reliably quantified by averaging over a large number of observations [7]. Other behaviors, such as those involved in learning and memory, can only be reliably assessed after a period of training.

In mice, several visually-evoked physiologic and behavioral responses have been used to assess motor function, cognition, and memory, as well as visual system function itself. In anesthetized mice, the light response of the outer retina, including the separate contributions of rod and cone systems, can be quantified by electroretinography (ERG)[8], [9]; and the strength of the retina-derived signal in the brain can be quantified with visually evoked potentials (VEPs)[8]. A relatively crude test of visual system function involves manually scoring the reflexive head turning that is elicited when an animal is placed in the center of a slowly rotating drum, a response that helps to stabilize the image of the drum on the retina [10], [11]. In awake and behaving mice, swimming tests guided by visual targets along the wall of a circular tank (the Morris water maze) have been used to measure spatial memory [12], two-way forced choice swimming tests have been used to measure visual acuity [13], and three-way forced choice tests with a food reward have been used to measure chromatic discrimination [14].

Image stabilization, noted above in the context of the head turning reflex, is predominantly mediated by two types of oculomotor responses: the optokinetic reflex (OKR; also called optokinetic nystagmus or OKN) and the vestibulo-ocular reflex (VOR)[15], [16]. The OKR is induced when the entire visual scene drifts across the retina, eliciting eye rotation in the same direction and at a velocity that minimizes the motion of the image on the retina. Steady eye rotation in the direction of stimulus motion is periodically interrupted by rapid rotations in the opposite direction (the quick phases or saccades), which reset the position of the eye for a new period of steady rotation. The VOR is an analogous response to head movement, with input coming from the vestibular system rather than the retina. Normally, the OKR and VOR work together to ensure image stabilization on the retina over a wide range of head and body motions.

Both the OKR and the VOR are largely controlled by subcortical circuits: the OKR is controlled by neurons in the retina, diencephalon and midbrain (the accessory optic system), pons, and dorsal medulla, and the VOR is controlled by neurons in the labyrinth of the inner ear, midbrain, pons, dorsal medulla, and cerebellum [16], [17]. In foveate animals, such as primates, eye movements that bring the object of regard onto the fovea add an additional layer of complexity and are controlled largely by the cerebral cortex [16].

In considering the neurologic assessment of mice, it would be useful to work with a stimulus-response paradigm that (1) is simple and rapid, (2) can be induced repeatedly with minimal fatigue or adaptation, (3) requires no training, (4) assesses the function of diverse CNS circuits, and (5) produces an electronic record that is readily and objectively quantifiable. For larger mammals, including humans, it has long been appreciated that these criteria are met by the OKR and the VOR [16], [18], [19]. While mouse eye movements have been studied by a number oculomotor research groups [15], [20]–[26], and oculomotor phenotypes have been characterized in several mutant or experimentally manipulated lines of mice [21], [27]–[30], oculomotor testing is rarely used by the wider neuroscience community. Indeed, there is no mention of it in the standard reference book on mouse behavioral phenotyping [6] or in standard compendia of mouse neurologic tests [31]. One barrier to the wider use of mouse oculomotor testing, as currently practiced, is its relatively low throughput; as a consequence, it has not been included among the neurologic tests used in conjunction with any of the large scale chemical mutagenesis screens [32], [33]. With the goal of making the mouse OKR a more accessible, versatile, and rapid test of CNS function, the present study describes a series of modifications of the test apparatus, visual stimulus, and data analysis, and applies these approaches to the quantification of genetic variation and CNS drug action.

## Results

### Measuring and scoring the murine OKR

In rodents, the lateral placement of the eyes provides a visual field that subtends ∼270° in the horizontal plane. To deliver an OKR stimulus that encompasses so large a visual field, the most common approach is to place the animal in the center of a vertical cylinder that is painted on its inner surface with vertical black and white stripes. The cylinder rotates around its axis and the OKR is monitored either by (1) tracking the orientation of a scleral search coil (a magnetic ring that is fixed to the outer surface of the eye)[24], [34]; or (2) using an infrared video camera to view, through a transparent circumferential slit in the cylinder, the position of the pupil [21], [26], [34]. One variation on the rotating cylinder method uses a non-rotating cylinder illuminated from above by light that passes through numerous small holes in a slowly rotating screen; the resulting pattern of light and shadow rotates around the inner face of the cylinder [35]. Another approach to creating a rotating stimulus uses a square stimulus chamber, the walls of which consist of four computer screens that display continuously moving vertical black and white stripes; this design has been used in the context of the head tracking optomotor response [11].

**Figure 1:**
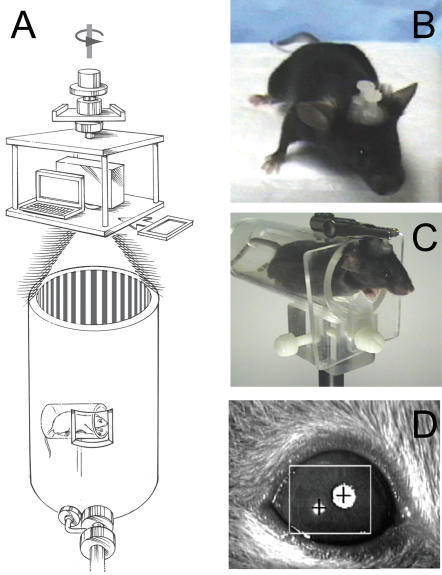
OKR apparatus and testing arrangement. (A) The mouse is held in a horizontal acrylic cylinder within a large cylindrical drum. A small transparent zone in the wall of the drum allows an infrared (IR) light source and camera (bottom, in foreground) to monitor eye movements. The stimulus is controlled by a laptop computer and a projector, both of which sit approximately two meters above the drum on a rotating table that is controlled by a variable speed motor (not shown). Neutral density or chromatic filters can be placed in the light path (rectangle, at right). (B) A headposted mouse with two plastic nuts set along the anterior posterior axis. (C) A headposted mouse in position for OKR measurements rests in the acrylic cylinder with its head protruding and immobilized by an alligator clip. (D) IR image of a mouse eye showing the pupil (large upper right white circle with centered cross) and the corneal reflection (lower left white circle with centered cross). The ISCAN software has assigned the IR sink as the pupil and the IR peak as the corneal reflection.

The OKR stimulus methods based on physical rotation of a cylinder or screen have the virtue that image motion is uniform and continuous, but they lack the flexibility associated with electronic stimulus presentation. By contrast, the use of multiple computer screens to form the walls of a stimulus chamber achieves the flexibility associated with electronic stimulus presentation but it imposes nonmoving borders at the interface between adjacent computer screens and it requires that visual stimuli be in the form of a movie, with an attendant increase in programming complexity. To combine the most favorable attributes of these two approaches, we designed and built an OKR apparatus in which a projector and laptop computer are mounted on a motorized rotating stage suspended 2 meters above the floor, with the optical axis of the projector coincident with both the axis of rotation of the stage and the axis of a white cylindrical test chamber at floor level (Figure 1A). A radially symmetric image on the computer screen (e.g. alternating black and white pie wedges) appears as a series of vertical lines when projected onto the cylindrical wall of the test chamber. The intensity of the image is varied either computationally or by inserting neutral density filters in front of the projector. A small window in the wall of the test chamber permits an infrared light source and video camera to image the eye of an awake and head posted mouse (Figure 1B–D).

Commercial software (ISCAN) was used to monitor the positions of (1) the pupil, the principal sink for both visible and infrared radiation, and (2) the point of reflection of incident infrared radiation at the surface of the cornea. For relatively small eye movements, the distance between the center of the pupil and the corneal reflection is roughly proportional to a frontal projection of the angular deflection of the eye. An empirical approach to determining this proportionality can be found in Sakatani and Isa [26]. This proportionality would be constant (and easily calculated) if (1) the eye was a perfect sphere that rotated about its center and (2) the pupil was located at the surface of the sphere. As discussed by Stahl et al. [34], neither of these conditions apply, but in practice the proportionality is still reasonably constant over different angles of deflection because the two principal deviations from the ideal case–namely, (1) the rotation of the eye around a point behind the center of the globe, and (2) the position of the pupil behind the corneal surface–produce largely compensating errors. In the interests of simplifying the OKR analysis, we show here only the horizontal distance between the pupil and the corneal reflection, referred to hereafter as “eye position”, without calculating the angle of eye rotation per se.

**Figure 2:**
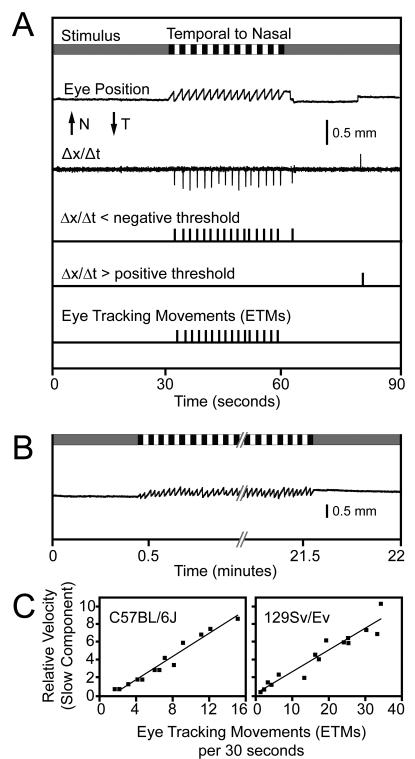
Stimulus, response, and data analysis during an OKR recording session with a wildtype mouse. (A) From top to bottom, the following features are shown. The schematic of the visual stimulus over a 90 second period represents: (1) a uniform grey during the first and last 30 second of the recording period, and (2) a pattern of black and white vertical stripes (each stripe subtending 4°) rotating at 5° per second in a temporal to nasal direction (with respect to the eye that is imaged) during the middle 30 seconds of the recording period. “Eye Position” indicates the horizontal difference between the center of the pupil and the center of the corneal reflection which shows a slow and uniform temporal-to-nasal motion interrupted approximately every two seconds by a rapid nasal-to-temporal saccade. N, nasal; T, temporal. The first derivative of eye position is shown above a pair of traces that indicate the times at which the first derivative exhibits an excursion below or above negative or positive thresholds, respectively. At the bottom are the assignments of discrete eye-tracking movements (ETMs) during the 30 second stimulus interval scored by inspection of eye position and its first derivative; the rightmost suprathreshold negative excursion on the first derivative plot is not counted as an ETM because it occurred outside of the 30 second stimulus interval. An identical ETM assignment was made by NeuralWorks Predict Version 3.13, a neural network (NeuralWare, Carnegie PA). (B) OKR over a continuous 21 minute period of rotating vertical black and white stripes shows essentially no adaptation. (C) ETMs per 30 second interval vs. the relative velocity of the slow component for C57BL/6J and 129/SvEv. Each data point is derived from a single 30-second stimulus interval obtained from the experimental data presented in Figure 5. A best fitting straight line is superimposed.

The OKR offers only a few parameters for quantitative evaluation, including the number of eye tracking movements (ETMs) per unit time and the gain (the angular velocity of the eye relative to the angular velocity of the stimulus). Here we define an ETM as one slow tracking movement followed by one saccade, and we quantify the ETMs by counting the number of saccades. In animals that lack a fovea, such as mice, the gain is typically less than unity, with the result that the image is not fully stabilized on the retina. We have focused on the number of ETMs per unit time because this parameter is relatively insensitive to changes in the shape of the slow component of the OKR, and it is easily quantified by automated procedures based on the first derivative of eye position with respect to time or with a neural network (Figure 2A and data not shown); it is also easily quantified by visual inspection. We note that the highly uniform time course and amplitude of the OKR in 129/SvEv mice is more readily scored than the somewhat variable OKR in C57BL/6J mice (see below for a comparison between these two strains; see also ref. 36 for an analysis of spontaneous eye movements in C57BL/6J mice). Artifacts due to eye blinks or to spontaneous eye movements not related to the OKR can generally be recognized by the presence of rapid movement in the “wrong” direction, i.e. the same direction as the rotating stimulus. A simple algorithm eliminates many of these artifacts by discounting all supra-threshold first derivative excursions in the “correct” direction that occur within 470 msecs of a supra-threshold excursion in the “wrong” direction.

The OKR reaches its steady state form within one second of the onset of stimulus motion and it shows no adaptation over more than 20 minutes of continuous stimulation, an interval that encompasses over 600 ETMs (Figure 2B). Related to this observation, Iwashita et al. [23] found that C57BL/6J mice subject to two hours of continuous oscillatory visual stimulation exhibited a modest improvement in OKR performance over time, with a slow increase in gain from 0.4 to 0.6 and a slow decrease in phase lag from 15° to 8°. As one would predict from visual inspection, the frequency of ETMs is roughly proportional to the average velocity of the slow component (Figure 2C).

Several research groups have studied mouse oculomotor behavior using oscillating stimuli under conditions that induce only the slow (tracking) component of the OKR [15], [26]. In the present study we have chosen to induce the OKR with a continuously rotating stimulus, thereby inducing both slow (tracking) and fast (resetting) components. This more complex response permits an assessment of changes in the frequency, amplitude, shape, and regularity of the alternating fast and slow components.

### Photoreceptor types responsible for the murine OKR

**Figure 3:**
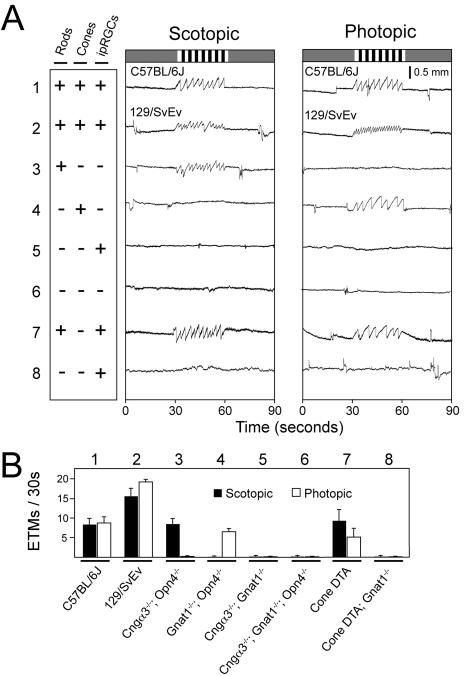
Photoreceptor subtypes subserving the scotopic and photopic OKR. (A) Scotopic (0.3 average lux) and photopic (200 average lux) stimuli consisting of the standard rotating black and white vertical stripes were presented to mice with the indicated functional (+) or silenced/absent (−) photoreceptors. ipRGC, intrinsically photosensitive retinal ganglion cells. Genotypes are shown beneath panel (B). The photopic stimulus is the same as the standard stimulus shown in Figure 2; the scotopic version was created from it by placing neutral density filters in front of the projector. 129/SvEv and C57BL/6J lines (rows 1 and 2) show characteristically different numbers of ETMs/second; lines with targeted gene mutations (rows 3–6 and 8) are maintained on mixed C57BL/6J×129/SvEv backgrounds. (B) Quantification of ETMs/30 seconds for the 8 lines of mice tested in panel (A). Mean +/− standard deviation for 5–12 mice per line and >31 30-second stimulus intervals per line.

As a test of the sensitivity and specificity of the OKR to variations in underlying physiologic parameters, we systematically explored the response to scotopic or photopic light levels (average stimulus intensities of 0.3 lux or 200 lux, respectively) in a variety of mutant mice in which rods, cones, or intrinsically photosensitive retinal ganglion cells (ipRGCs) were eliminated or inactivated individually or in various combinations (Figure 3). For these experiments, rod, cone or ipRGC function was individually eliminated by targeted disruption of the genes coding for, respectively, rod transducin-alpha (*Gnat1*)[37], one of the two cone cyclic nucleotide gated channel subunits (*Cngα3*)[38], or melanopsin, the ipRGC-specific opsin (*Opn4*)[39]. We also used a transgene that expresses an attenuated diphtheria toxin A chain under the control of a human L cone opsin promoter and enhancer to ablate all medium wavelength (M) cones and almost all short wavelength (S) cones (*Cone DTA*)[40].

Mice possessing functional rods but lacking functional cones and ipRGCs show an OKR only at scotopic light levels (Figure 3A, line 3), whereas mice possessing functional cones but lacking functional rods or ipRGCs show an OKR only at photopic light levels (Figure 3A, line 4). Mice lacking both rod and cone function, with or without functional ipRGCs, show no OKR at any light level (Figure 3A, lines 5 and 6), consistent with the non-image forming nature of the ipRGC signal [41] and previous optomotor behavioral experiments [42]. In testing the OKR under photopic light levels, we discovered that rods continue to drive the OKR unless pupil constriction is blocked either pharmacologically or by genetically eliminating ipRGC function (as shown in Figure 3A, line 3), presumably because pupil constriction decreases the light intensity at the retina to the point where the rod system remains active. Consistent with this observation, ablating cones with diphtheria toxin, while leaving the ipRGC system intact, does not eliminate the photopic OKR (Figure 3A, line 7) unless rod function is eliminated (Figure 3A, line 8) or the pupils are dilated pharmacologically (data not shown).

As a further measure of the sensitivity of the OKR to underlying retinal physiology, we determined the spectral sensitivity of the cone-driven OKR in mice that differed with respect to the spectral sensitivity of their longer wavelength cone pigment. Wild type mice express two types of cone pigment: an S pigment, with maximal absorption at 360 nm, and an M pigment, with maximal absorption at 510 nm [43]. The M pigment is encoded on the X-chromosome. In earlier work, we genetically engineered a line of knock-in mice that express a human L pigment, with maximal absorption at 556 nm, in place of the mouse M pigment [14], [44]. In the present experiment, we have compared the strength of the OKR among mice expressing (in addition to the S-pigment): (1) only the mouse M pigment, (2) only the human L pigment, or (3) both M and L pigments. For the third class of mice, X-inactivation in heterozygous females creates a fine-grained mosaic of M and L cones across the retina [44]. To insure that the OKR reflects only cone function, these experiments were performed on a rod transducin alpha knockout genetic background (*Gnat1^−/−^*). A further simplification arises from the relative insensitivity of the UV-sensitive S pigment to the visible wavelengths that dominate the output of the projector. Therefore, under these experimental conditions, the OKR should be driven almost exclusively by the output of M and/or L cones.

For the chromatic OKR experiment, a pattern of alternating colored and grey stripes is presented. The intensity, hue, and saturation of the colored stripe are held constant, while the lightness of the grey stripe is varied in 10% increments from white to black. For a mouse expressing only M pigment, the number of photons captured per unit time from the grey stripe equals, at some point along a continuum of grey intensities, the number captured from the colored stripe. At that point, the OKR is extinguished, and, therefore, this grey intensity represents the null point for the response. A similar argument applies to mice expressing only the L pigment. This experimental design closely resembles an optokinetic test developed by Teller and Palmer [45] to assess red/green color vision in human infants.

In classical color vision psychophysics, gratings and filters permit the production of chromatic stimuli with wavelength compositions that are narrow and adjustable. By contrast, the three channels of the projector provide stimuli with spectral compositions that are fixed and relatively broad. Even for lights that appear to the human eye to have nearly pure hue and full saturation, direct spectroradiometer measurement shows a relatively complex spectral composition (Figure S1).

**Figure 4:**
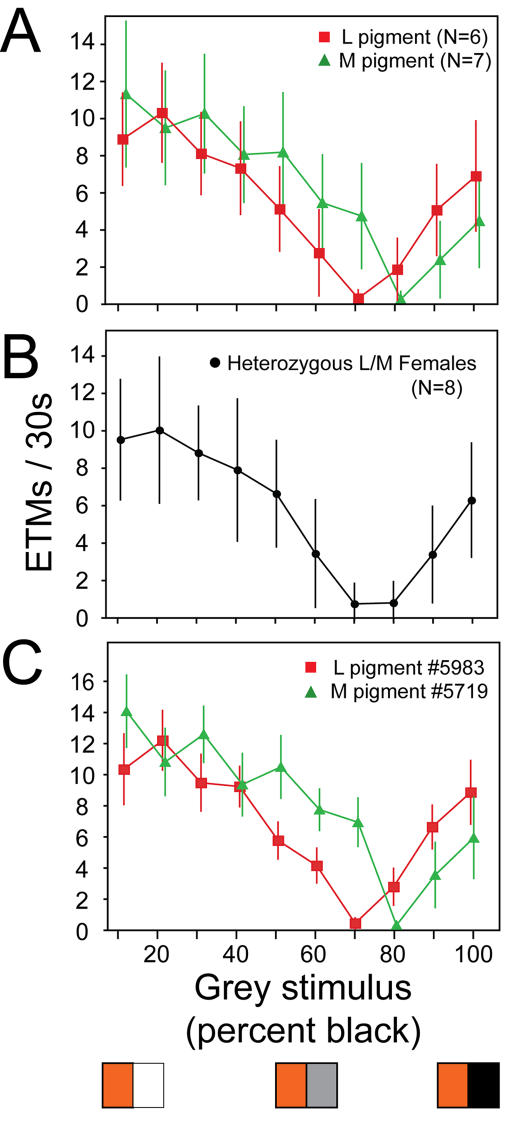
Chromatic sensitivity of the OKR in mice with different cone photopigments. (A) Number of ETMs (mean +/− standard deviation) per 30-second interval for each of ten stimuli consisting of alternating orange and grey vertical stripes, with each stripe subtending 4°. The image was rotated at 5° per second. For the ten different stimuli, the orange intensity was held constant and the grey intensity was varied in 10% intensity steps from white (stimulus 1) to black (stimulus 10) as shown by the three representative panels at the bottom of the figure. The responses are from seven Gnat1^−/−^;M pigment mice and six Gnat1^−/−^;L pigment mice. Each point represents 9–20 30-second stimulus intervals per condition. The interpolated grey intensity that yields the fewest ETMs per 30 seconds provides the best estimate of the psychophysical null point. (B) As for panel (A) but averaged data from eight *Gnat1^−/−^*;M pigment/L pigment heterozygous female mice. (C) As for panel (A), but the responses are from a single *Gnat1^−/−^*;M pigment mouse and a single *Gnat1^−/−^*;L pigment mouse.

Despite the limited chromatic purity of the stimulus, the OKR experiment shows a clear distinction among mice expressing different cone pigments (Figure 4). As predicted by the greater sensitivity of the human L pigment to longer wavelengths, when the variable grey panels are paired with an orange stimulus (i.e. one that is more efficiently captured by the L than the M pigment) the null point for the mouse expressing L pigment occurs with a ∼10% brighter grey than does the null point for the mouse expressing M pigment (Figure 4A). Moreover, the null points can be reliably determined for individual mice (Figure 4C). Compared to homozygotes, L/M heterozygotes show a broader and shallower zone of minimal OKR response (Figure 4B), suggesting that, to first approximation, the OKR in heterozygotes is driven by signals averaged from both L and M cones.

### Stimulus-response parameters for the murine OKR

To compare the basic response properties of the OKR between C57BL/6J and 129/SvEv, we have analyzed the effect of contrast, spatial frequency, angular velocity, monocular vs. binocular presentation, and direction of rotation, in each case quantifying the OKR response by determining the average number of ETMs per 30-second stimulus interval. Some of these parameters have been analyzed previously with respect to the gain and phase of the OKR using oscillatory stimuli that elicit only the slow phase of the OKR, and generally only with C57BL/6J mice [15], [22]–[25]. Contrast and spatial frequency thresholds have been measured by visual inspection of head and body turning in freely moving mice (the optomotor response)[11]. In the present experiments, we have compared C57BL/6J, the most frequently used strain for behavioral testing [6], and 129/SvEv, a substrain closely related to the 129/Sv substrain from which the most commonly used embryonic stem (ES) cell lines are derived [46] and one that is often used to explore the effect of genetic background on phenotypes associated with targeted genetic alterations.

**Figure 5:**
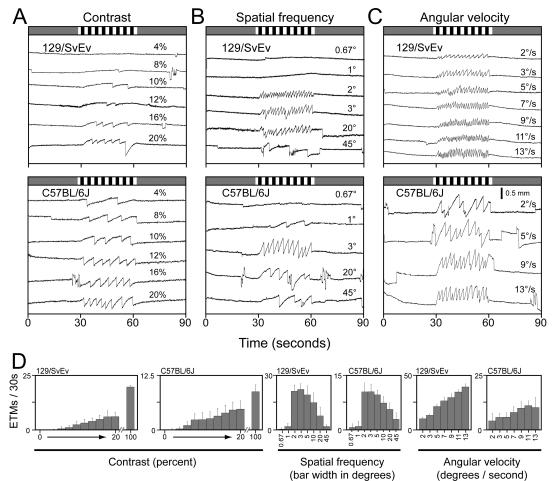
The sensitivity of the OKR in 129/SvEv and C57BL/6J mice to variations in contrast, spatial frequency, and angular velocity. For each of the three stimulus parameters examined, examples of OKR recordings are shown for both 129/SvEv and C57BL/6J (upper panels) and the mean +/− standard deviations of those responses for 4–9 mice per genotype and 11–46 30-second stimulus intervals (lower panels). Unless otherwise indicated the stimulus parameters match those shown in Figure 2. (A) Contrast sensitivity. The standard black and white striped stimulus (Figure 2; 100% contrast) was modified by producing a weighted average between the black and white stripes and a standard grey with a luminance midway between that of the black and white stripes. This operation creates stripes with contrasts ranging from 0% to 100% without changing the average luminance of the stimulus. (B) Spatial frequency. The width of each black and white stripe varied from 0.67° to 45°. (C) Angular velocity. The angular velocity of the standard stimulus varied from 2° per second to 13° per second.

Decreasing the contrast of the light and dark stripes progressively reduces the strength of the OKR with a threshold of detection (arbitrarily defined as greater than one ETM per 30 second interval) for C57BL/6J mice at ∼2% contrast and for 129/SvEv at ∼6% contrast (Figure 5A and Figure S2). The optimal spatial frequency for both C57BL/6J and 129/SvEv occurs at a stripe width of ∼3°, which subtends ∼90 um at the retina if we take 1.69 mm as the average diameter of the murine eye (Figure 5B)[34]. This spatial resolution is marginally higher than the average receptive field diameter of murine retinal ganglion cells, estimated to be ∼130 um [44] or ∼200 um [47]. C57BL/6J mice are somewhat more sensitive than 129/SvEv to high spatial frequencies, as seen in comparing the OKR to 1° stripes. The OKR response to angular velocities between 2° and 13° per second shows that both strains have broad sensitivity with a roughly linear response over this range of velocities (Figure 5C). The series of OKR responses shown in Figure 5C also illustrates the characteristic saccade-to-saccade variability of the C57BL/6J OKR, a variability that becomes more pronounced with suboptimal stimuli. Spontaneous eye movements are also evident during the 30-second rest periods and are more frequently produced by C57BL/6J than 129/SvEv mice.

**Figure 6:**
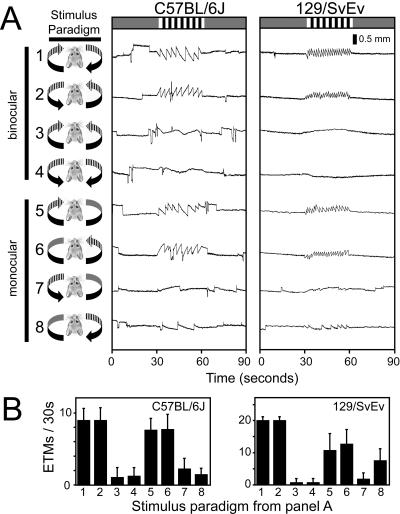
The sensitivity of the OKR in 129/SvEv and C57BL/6J mice to monocular or binocular rotation in temporal-to-nasal or nasal-to-temporal directions. (A) Left, schematic of the geometry of the stimulus. The OKR is always recorded from the right eye, and standard stimulus parameters are used (Figure 2). Rows 1 and 2 show responses to standard clockwise or counterclockwise rotation. Rows 3 and 4 show the nulling of mirror symmetric temporal-to-nasal or nasal-to-temporal stimuli. Rows 5 and 6 show the strong response to monocular stimuli in the temporal-to-nasal direction delivered to either the contralateral or ipsilateral eye. Rows 7 and 8 show the weak response to monocular stimuli in the nasal-to-temporal direction delivered to either the contralateral or ipsilateral eye. For monocular moving stimuli (rows 5–8), an intensity-matched uniform grey stimulus was delivered to the contralateral eye. (B) Quantifying the number of ETMs/30 seconds for the stimuli tested in panel (A). Mean +/− standard deviation for >6 mice for each strain and 25–39 30-second stimulus intervals per condition. N, nasal; T, temporal.

When the entire image rotates within the drum or, under natural conditions, when a horizontal head turning movement produces a uniform rotation of the entire visual world, the mouse experiences simultaneous nasal-to-temporal stimulus motion in one eye and temporal-to-nasal stimulus motion in the contralateral eye. The resulting robust OKR is taken as the point of reference in assessing monocular nasal-to-temporal and temporal-to-nasal stimuli, (Figure 6A, rows 1 and 2). As previously described in rabbits and rats-two mammals that, like mice, have very little binocular vision–the OKR under conditions of uniform rotation is not driven equally by the two eyes [35], [48]. Instead, monocular temporal-to-nasal motion induces a strong OKR in both eyes, whereas monocular nasal-to-temporal motion induces only a weak OKR (compare Figure 6A, lines 5 and 6 vs. lines 7 and 8). Consistent with a conjugate response, the OKR is nulled by symmetric binocular temporal-to-nasal or nasal-to-temporal motion (delivered by projecting a movie, since mirror symmetric stimuli cannot be produced by rotating the projector; Figure 6A, lines 3 and 4).

### Polygenic inheritance of strain differences in the OKR

Substantial variations have been reported in OKR response amplitudes in various inbred strains of mice [20], [21]. In the present study, 129/SvEv mice showed a smaller saccade amplitude, a greater ETM frequency, and a greater uniformity of the OKR relative to C57BL/6J (Figures 3, 5, and 6). Additionally, 129/Sv has a significant phase defect in an oscillatory visual stimulus paradigm relative to C57BL/6J [49].

The strain difference in OKR amplitude could conceivably arise from a constraint on the mobility of the eye in 129/SvEv mice. Arguing against this possibility, we saw no obvious anatomic differences between these strains when comparing magnetic resonance images of the intact eye and orbit at a spatial resolution between 55 um and 100 um (data not shown). Moreover, spontaneous eye movements, although rare in 129/SvEv mice relative to C57BL/6J (Figure 5), are occasionally of large amplitude, indicating that the 129/SvEv orbit can accommodate large amplitude eye movements.

**Figure 7:**
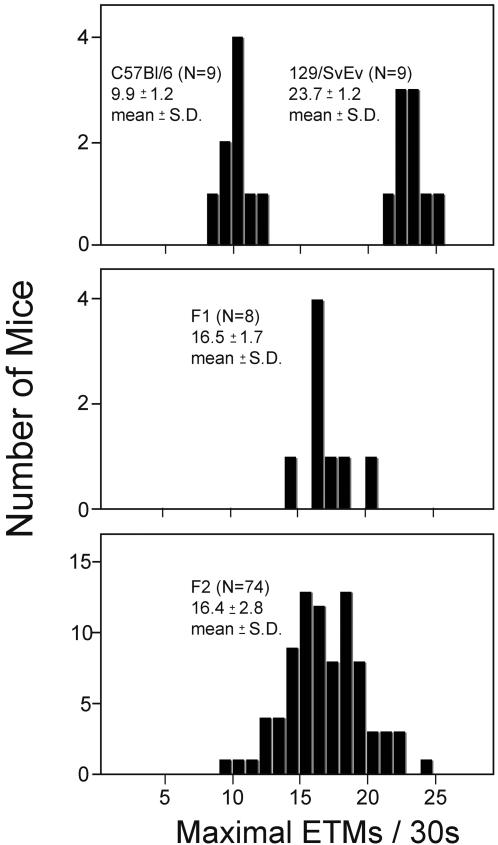
Polygenic inheritance of the number of ETMs per 30-second stimulus interval. The maximal number of ETMs per 30-second stimulus interval, determined from 16 trials per mouse, was measured for cohorts of C57BL/6J, 129/SvEv, C57BL/6J×129/SvEv F1 hybrids, and F2 progeny of the F1 hybrids. The number of animals tested, and the means and standard deviations are indicated for each distribution.

To assess the mode of inheritance of saccade frequency, nine C57BL/6J mice, nine 129/SvEv mice, eight C57BL/6J×129/SvEv F1 mice, and 74 F2 mice (derived by intercrossing the F1 mice) were tested to determine the maximal number of ETMs per 30-second interval (from among 16 such intervals per mouse) using the standard OKR stimulus (Figure 7). For this analysis, we have used the maximal number of ETMs rather than the mean because the standard deviation of the maximal number is somewhat smaller than the standard deviation of the mean. The resulting distributions demonstrate that this characteristic is inherited as a polygenic trait, as judged by the relatively narrow distribution of ETMs in the F2 generation. If we make the simplifying assumption that the relevant genes segregate randomly, are codominant, and are of equal effect, then the number of genes is readily estimated by comparing the observed distribution of ETMs in the F2 cohort to the calculated distribution of C57BL/6J and 129/SvEv alleles in the F2 generation [50]. This comparison predicts that at least six genes control the difference in ETM frequencies in these two strains. At present, the nature of these genetic differences remains to be determined. As a practical matter, this experiment also indicates that targeted mouse mutants maintained on a mixed C57BL×129/Sv background will likely display individual variation in the OKR due to randomly segregating genetic background differences unless these differences are eliminated by backcrossing to one of the pure parental lines.

### The OKR as a quantitative measure of CNS drug action

At present, one of the major challenges in CNS drug development is the establishment of quantitative and high throughput animal tests that can be used to narrow a large numbers of candidate compounds for clinical testing. Although eye movements are not generally studied as an intended physiologic target of CNS drug action, in humans many drugs that act on the CNS modulate eye movements, including sedatives, anti-psychotics, and anti-depressants [16]. These observations suggest that the OKR response in mice could be used as a tool to characterize CNS drug action.

**Figure 8:**
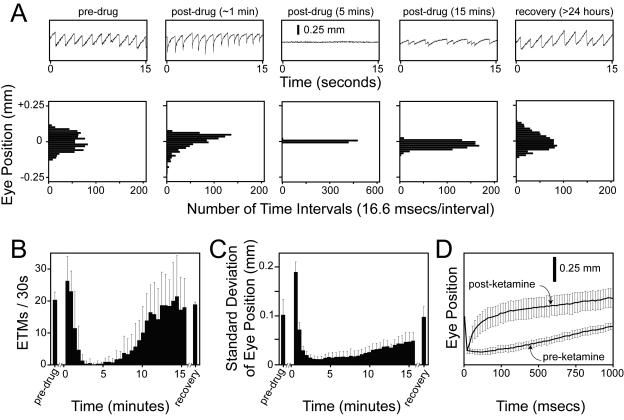
Time course of alterations in the OKR following IP ketamine injection into 129/SvEv mice. (A) Each 15 second ETM record (upper panels), obtained at the indicated time before or after a 75 mg/kg IP ketamine injection, is shown above a histogram of eye positions during the same 15 second interval (lower panels). The histograms were calculated using eye position values sampled at 60 Hz and processed as described under Experimental Procedures. (B) Average and standard deviation of the number of ETMs per 30-second interval following IP ketamine at time 0 (N = 5 mice). For panels (B) and (C), the first 30-second interval following ketamine injection begins 15–20 seconds after injection. (C) Average and standard deviation of the standard deviation of eye position for each 30-second interval following IP ketamine at time 0 (N = 5 mice). (D) A systematic distortion in the shape of the slow component of the OKR between 45 and 90 seconds after ketamine injection (“post-ketamine”). The left-most data points in each curve correspond to the start of the slow phase. Note the enlarged time scale on the horizontal axis. (N = 22–24 ETMs/averaged curve, obtained from 3 mice.)

As an initial test of this idea, we have examined the time course of ketamine action on the mouse OKR following intraperitoneal (IP) delivery (Figure 8). Five intervals from a single experiment are shown in the upper panels of Figure 8A. The histograms in the lower panels of Figure 8A show the distribution of eye positions for the same intervals. The number of ETMs and the standard deviation of the eye position distributions from consecutive 30 second intervals are plotted in Figure 8B and 8C for the pre-injection, post-injection, and recovery stages of the experiment. In control experiments, IP injection of saline produced no changes in the OKR.

Ketamine is a NMDA receptor antagonist that acts rapidly as a sedative and anesthetic [51]. When it is delivered IP at 75 mg/kg, the mouse responds with the following stereotyped sequence of OKR changes: (1) during an initial period of 1–2 minutes, each ETM shows a rapid rebound at the beginning of the slow phase, an effect that could arise from a defect in the oculomotor neural integrator [52], [53] (Figure 8A, second panel, and Figure 8D); (2) over the next ∼5 minutes, there is a diminution in OKR amplitude with complete or nearly complete elimination of the OKR (Figure 8A, third panel); (3) over tens of minutes, the OKR recovers but shows saccades of highly variable timing and amplitude (Figure 8A, fourth panel); and (4) by 24 hours later there is a full recovery (Figure 8A, fifth panel). To quantify this response with a fully automated procedure, we counted ETMs using a first derivative thresholding operation (Figures 2 and 8B) and determined the standard deviation of the distribution of the eye position (Figure 8C), a combination that gives a good overall picture of the ketamine response. More complex procedures could be devised to quantify variability in ETM shape, timing, and amplitude.

## Discussion

In the present work we (1) describe the design of a novel OKR test apparatus in which a rotating computer-controlled projector delivers diverse and easily generated OKR stimuli, and (2) use infrared imaging to monitor the murine OKR and then quantify the number of ETMs per unit time in a rapid, objective, and reproducible manner. We have used this system to (1) define the basic OKR stimulus-response characteristics of C57BL/6J and 129/SvEv and of genetically engineered variants that lack one or more photoreceptor systems or that have an altered spectral sensitivity, (2) show that the characteristic difference in OKR frequency between C57BL/6J and 129/SvEv is inherited as a polygenic trait, and (3) demonstrate that the mouse OKR can be used as a sensitive and quantitative assay for drug action in the CNS. These experiments show that, despite its seeming simplicity, the OKR provides a rich record of CNS activity.

As demonstrated by our analysis of the polygenic inheritance of OKR differences between strains, OKR testing can be used to analyze dozens to hundreds of mice, generating data sets with many thousands of ETMs. Furthermore, the ketamine experiment demonstrates the high sensitivity and temporal resolution of the OKR in monitoring drug effects on CNS function. These examples suggest that the murine OKR could be usefully applied to a wide variety of investigations. For example, the OKR could be used to quantify drug-induced sedation or arousal, or to monitor the progression or the therapeutic response of neurodegenerative disease. The OKR is also well suited for quantifying the functional response to stem cell, pharmacologic, or genetic approaches aimed at restoring vision in the context of retinal degenerative diseases. In summary, the OKR can help meet the growing demand for functional neurologic testing as drug discovery programs generate a growing pipeline of preclinical drug candidates and as targeted and random mutagenesis technologies generate increasing numbers of mice with CNS defects.

## Methods

### Animal Surgery

Mice were handled and housed in accordance with the Johns Hopkins University Animal Care and Use Protocols and IACUC guidelines. For headpost surgery, instruments were autoclaved, a sterile environment was prepared, and mice were anesthesized using a ketamine/xylazine mixture. Ophthalmic ointment was applied to the eyes during surgery to prevent corneal drying and lens opacification, a side-effect of ketamine/xylazine anesthesia. Once the mouse was areflexive to paw pinch, povidone-iodine ointment was used to sterilize the top of the head, and an incision was made to expose the cranium. 0.5 ml of 3% hydrogen peroxide was applied to the skull to remove soft tissue covering the bone. Four holes were drilled into the skull, and four 0.93 mm diameter metal screws (Plastics One; Roanoke, VA; Part Number: #8L010121202F) were inserted to provide support. Dental cement (a 1:1 mixture of methyl methacrylate and diethyl phthalate; Lang Dental; Wheeling,IL, Part Number: 60090) was mixed and added drop-wise to the top of the skull to cover the metal screws. Two 1/8 inch nylon screws and hex nuts (width across flats: 0.180 inch; thickness: 0.070 inch; thread size: 2–56; Small Parts Inc; Miami Lakes, FL, Part Numbers: MN-0256-04B and HNN-0256-M) were inserted into the solidifying dental cement along the midline such that each nut and the distal half of each screw was embedded in the resin while the head of the screw protruded upward (Figure 1B). Following the ∼15 minute surgery, the mice were placed on a 37°–42°C heating pad. For sequential surgeries, instruments were sterilized between mice by immersion in Cidex (Advanced Sterilization Products; Miami, FL; Part Number: 2245). The mice typically awoke 30–60 minutes after surgery and were monitored for feeding, grooming, and gait. Buprenorephine (0.5mg/kg) was delivered as needed for pain or discomfort. OKRs were recorded >48 hours after surgery.

### OKR apparatus

Stimuli were projected vertically into a clear plastic drum (29.5 cm diameter×61 cm height) lined with white reflective poster paper using a Mitsubishi SL2U projector mounted on a rotating aluminum stage 91 cm above the top of the drum. A headposted mouse centered within the cylinder and 20 cm below the upper edge of the cylinder sees the rotating stimulus throughout the visual field except for a vertically centered cone subtending ∼35 degrees. An electric motor (Dayton; Niles, IL; Part Number: 4Z525A) controlled by a DC Speed Control (Dayton) rotated the stage. A rotating electrical adaptor (Moog; Blacksburg, Virginia) supplied power to the projector. To deliver scotopic stimuli, neutral density filters (ThorLabs; Newton, NJ) were placed in the light path using a holder fixed to the bottom of the rotating aluminum stage.

### Visual stimuli

Visual stimuli were designed in Adobe Photoshop and displayed using Microsoft PowerPoint. To create vertical stripes on the walls of the test cylinder, we projected a circularly symmetric series of alternating black and white sectors (i.e. pie wedges) centered on the axis of the cylinder. Unless otherwise stated, OKR stimuli were presented as alternating 30-second intervals of moving black and white stripes and of a uniform grey. The geometry of the apparatus dictates that the projected image exhibits a progressive change in focus and a diminution in intensity from the top to the bottom of the cylinder. As both of these variations are barely noticeable to a human observer, we have not attempted to correct them. The uniform grey illuminant used during the rest periods was set to an intensity that delivered the same number of photons as the average of the black and white striped OKR stimuli. As expected, the pupil neither constricts nor dilates during the transitions between rest and stimulus intervals. For monocular stimulation, an opaque panel was placed over one-half of the test cylinder.

### OKR measurements and data analysis

For genotypes that eliminate pupil constriction (*Cngα3^+/+^*; *Gnat1^−/−^*; *Opn4^−/−^* and *Cngα3^−/−^*; *Gnat1^−/−^*; *Opn4^−/−^*), 5–10 µl of 3% pilocarpine hydrochloride or 3% carbamoylcholine chloride was applied to the eye to induce constriction. Pupil constriction improves the accuracy of the calculation that identifies the center of the pupil. In mice with other genotypes, the intrinsic pupil constriction with photopic stimuli was sufficient for accurate measurements of pupil position. With scotopic stimuli, all mice were treated with a constricting agent.

To collect OKR data, the eye was imaged through a small hole cut in the poster paper using infrared illumination and an ISCAN (Burlington, MA) infrared video camera. ISCAN software locates the horizontal and vertical coordinates of the calculated centers of the corneal reflection and the pupil, and creates a time series (sampled at 60 Hz) of the difference between these two points along the horizontal and vertical axes. The data were stored as a Microsoft Excel file, and prior to data analysis the eye position data file was edited by discarding excursions >1.15 mm from the mean eye position. Larger excursions arise from errors in the automated assignment of pupil location. Time derivatives and thresholding operations were performed in Excel. ETM assignments were made by visual inspection, by thresholding the first derivative of eye position, or by NeuralWorks Predict Version 3.13 (NeuralWare, Carnegie PA).

### Ketamine experiments

For each mouse, the OKR was recorded before and >24 hours after drug exposure using the standard 30 seconds on/30 seconds off OKR stimulus paradigm (Figure 2) with alternating clockwise and counterclockwise rotation. To initiate an experiment, 75 mg/kg ketamine was administered by IP injection, and the mouse was immediately transferred to the recording chamber to begin measuring the OKR. A strong optokinetic stimulus (4° wide black and white stripes rotating at 5°/second) was continuously displayed during the 15–20 minute recording session. The OKR records were subsequently divided into 30 second intervals, and the following parameters were calculated for each 30 second interval: (1) the number of ETMs, as determined by automated thresholding of the first derivative (Figure 2), and (2) the distribution and standard deviation of eye position (1800 data points per interval).

The eye position data file was subject to a local smoothing procedure with the following formula [where x_n_ is the n^th^ eye position measurement (sampled at 60 Hz) and τ is set to 0.8]:
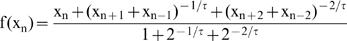



To calculate the standard deviation of eye position in 30 second intervals, the eye position at each time point was further modified by subtracting from it the average eye position over a three second moving window centered on that time point, an operation that largely compensates for any slow background drift in eye orientation. To evaluate drug-induced changes in the slow component of the OKR, individual ETMs were aligned and averaged beginning with the first time point after the end of the fast component (Figure 8D).

## Supporting Information

Figure S1 Spectral composition and relative intensities of OKR stimuli used for chromatic vs. gray scale experiments. Spectroradiometer measurements were made of the light reflected from the inner wall of the testing cylinder at the position of the mouse holder, when the LCD projector illuminated the cylinder with the indicated stimulus lights.
(9.39 MB TIF)

Figure S2 Relative gain in the slow component of the OKR as a function of percent contrast between the black and white stripes. Data are from the experiment shown in Figure 5A. Eye rotation is calculated based on a simplified model that places both the pupil image and the corneal reflections at the surface of the globe and assumes rotation about the center of a spherical globe (see Results section). A gain of 1.0 corresponds to an eye rotation that exactly tracks the stimulus.
(9.20 MB TIF)

## References

[1] Picciotto MR, Wickman K (1998). Using knockout and transgenic mice to study neurophysiology and behavior.. Physiol Rev.

[2] Brunner D, Nestler E, Leahy E (2002). In need of high-throughput behavioral systems.. Drug Discovery Today.

[3] Tecott LH, Nestler EJ (2004). Neurobehavioral assessment in the information age.. Nat Neurosci.

[4] Crabbe JC, Morris RG (2004). Festina lente: late-night thoughts on high-throughput screening of mouse behavior.. Nat Neurosci.

[5] Mancall EM (1982). Essentials of the Neurologic Examination (second edition)..

[6] Crawley JN (2000). What's Wrong With My Mouse? Behavioral Phenotyping of Transgenic and Knockout Mice..

[7] de Visser L, van den Bos R, Kuurman WW, Kas MJ, Spruijt BM (2006). Novel approach to the behavioural characterization of inbred mice: automated home cage observations.. Genes Brain Behav.

[8] Pinto LH, Enroth-Cugell C (2000). Tests of the mouse visual system.. Mamm Genome.

[9] Pinto LH, Invergo B, Shimomura K, Takahashi JS, Troy JB (2007). Interpretation of the mouse electroretinogram.. Doc Ophthalmol.

[10] Thaung C, Arnold K, Jackson IJ, Coffey PJ (2002). Presence of visual head tracking differentiates normal sighted from retinal degenerate mice.. Neurosci Lett.

[11] Douglas RM, Alam NM, Silver BD, McGill TJ, Tschetter WW (2005). Independent visual threshold measurements in the two eyes of freely moving rats and mice using a virtual-reality optokinetic system.. Vis Neurosci.

[12] Lipp HP, Wolfer DP (1998). Genetically modified mice and cognition.. Curr Opin Neurobiol.

[13] Prusky GT, West PW, Douglas RM (2000). Behavioral assessment of visual acuity in mice and rats.. Vision Res.

[14] Jacobs GH, Williams GA, Cahill H, Nathans J (2007). Emergence of novel color vision in mice engineered to express a human cone photopigment.. Science.

[15] Stahl JS (2004). Using eye movements to assess brain function in mice.. Vision Res.

[16] Leigh RJ, Zee DS (2006). The Neurology of Eye Movements (fourth edition)..

[17] Simpson JI (1984). The accessory optic system.. Annu Rev Neurosci.

[18] Dodge R (1903). Five types of eye movement in the horizontal meridian plane of the field of regard.. Am J Physiol.

[19] Alpern M, Davson H (1969). Movements of the eyes.. The Eye.

[20] Balkema GW, Mangini NJ, Pinto LH, Vanable JW (1984). Visually evoked eye movements in mouse mutants and inbred strains.. Inv Ophthalmol Vis Sci.

[21] Mangini NJ, Vanable JW, Williams MA, Pinto LH (1985). The optokinetic nystagmus and ocular pigmentation of hypopigmented mouse mutants.. J Comp Neurol.

[22] de Zeeuw CI, van Alphen AM, Koekkoek SK, Buharin E, Coesmans MP (1998). Recording eye movements in mice: a new approach to investigate the molecular basis of cerebellar control of motor learning and motor timing.. Otolaryngol Head Neck Surg.

[23] Iwashita M, Kanai R, Funabiki K, Matsuda K, Hirano T (2001). Dynamic properties, interactions and adaptive modifications of vestibulo-ocular reflex and optokinetic response in mice.. Neurosci Res.

[24] van Alphen AM, Stahl JS, de Zeeuw CI (2001). The dynamic characteristics of the mouse horizontal vestibulo-ocular and optokinetic response.. Brain Res.

[25] Faulstich BM, Onori KA, du Lac S (2004). Comparison of plasticity and development of mouse optokinetic and vestibulo-ocular reflexes suggests differential gain control mechanisms.. Vision Res.

[26] Sakatani T, Isa T (2004). PC-based high-speed video-oculography for measuring rapid eye movements in mice.. Neurosci Res.

[27] Iwakabe H, Katsura G, Ishibashi C, Nakanishi S (1997). Impairment of pupillary responses and optokinetic nystagmus in the mGluR6-deficient mouse.. Neuropharmacology.

[28] Kitazawa H, Katoh A, Yagi T, Nagao S (2000). Dynamic characteristics and adaptability of reflex eye movements of Fyn-kinase-deficient mice.. Neurosci Lett.

[29] Yoshida K, Watanabe D, Ishikane H, Tachibana M, Pastan I (2001). A key role of starburst amacrine cells in originating retinal directional selectivity and optokinetic eye movement.. Neuron.

[30] Stahl JS, James RA, Oommen BS, Hoebeek FE, de Zeeuw CI (2006). Eye movements of the murine P/Q calcium channel mutant tottering, and the impact of aging.. J Neurophysiol.

[31] Long JM (2006). Analysis of neural cell function in gene knockout mice: behavior.. Methods Enzymol.

[32] Moldin SO, Farmer ME, Chin HR, Battey JF (2001). Trans-NIH initiatives on mouse phenotyping and mutagenesis.. Mamm Genome.

[33] Vitaterna MH, Pinto LH, Takahashi JS (2006). Large-scale mutagenesis and phenotypic screens for the nervous system and behavior in mice.. Trends Neurosci.

[34] Stahl JS, van Alphen AM, de Zeeuw CI (2000). A comparison of video and magnetic search coil recordings of mouse eye movements.. J Neurosci Methods.

[35] Hess BJ, Precht W, Reber A, Cazin L (1985). Horizontal optokinetic ocular nystagmus in the pigmented rat.. Neuroscience.

[36] Sakatani T, Isa T (2007). Quantitative analysis of spontaneous saccade-like rapid eye movements in C57BL/6 mice.. Neurosci Res.

[37] Calvert PD, Krasnoperova NV, Lyubarsky AL, Isayama T, Nicolo M (2000). Phototransduction in transgenic mice after targeted deletion of the rod transducin alpha -subunit.. Proc Natl Acad Sci USA.

[38] Biel M, Seeliger M, Pfeifer A, Kohler K, Gerstner A (1999). Selective loss of cone function in mice lacking the cyclic nucleotide-gated channel CNG3.. Proc Natl Acad Sci USA.

[39] Lucas RJ, Hattar S, Takao M, Berson DM, Foster RG (2003). Diminished pupillary light reflex at high irradiances in melanopsin-knockout mice.. Science.

[40] Soucy E, Wang Y, Nirenberg S, Nathans J, Meister M (1998). A novel signaling pathway from rod photoreceptors to ganglion cells in mammalian retina.. Neuron.

[41] Hattar S, Lucas RJ, Mrosovsky N, Thompson S, Douglas RH (2003). Melanopsin and rod-cone photoreceptive systems account for all major accessory visual functions in mice.. Nature.

[42] Schmucker C, Seeliger M, Humphries P, Biel M, Schaeffel F (2005). Grating acuity at different luminances in wild-type mice and in mice lacking rod or cone function.. Invest Ophthalmol Vis Sci.

[43] Jacobs GH, Neitz J, Deegan JF (1991). Retinal receptors in rodents maximally sensitive to ultraviolet light.. Nature.

[44] Smallwood PM, Olveczky BP, Williams GL, Jacobs GH, Reese BE (2003). Genetically engineered mice with an additional class of cone photoreceptors: implications for the evolution of color vision.. Proc Natl Acad Sci USA.

[45] Teller DY, Palmer J (1996). Infant color vision: motion nulls for red/green vs luminance-modulated stimuli in infants and adults.. Vision Res.

[46] Simpson EM, Linder CC, Sargent EE, Davisson MT, Mobraaten LE (1997). Genetic variation among 129 substrains and its importance for targeted mutagenesis in mice.. Nat Genet.

[47] Balkema GW, Pinto LH (1982). Electrophysiology of retinal ganglion cells in the mouse: a study of a normally pigmented mouse and a congenic hypopigmentation mutant, Pearl.. J Neurophys.

[48] Collewijn H (1969). Optokinetic eye movements in the rabbit: input-output relations.. Vision Res.

[49] Katoh A, Kitazawa H, Itohara S, Nagao S (1998). Dynamic characteristics and adaptability of mouse vestibulo-ocular and optokinetic response eye movements and the role of the flocculo-olivary system revealed by chemical lesions.. Proc Natl Acad Sci USA.

[50] Cavalli-Sforza LL, Bodmer WF (1971). The Genetics of Human Populations..

[51] Marshall BE, Longnecker DE, Hardman JG, Limbird LE, Moloinoff PB, Ruddon RW, Gilman AG (1996). General Anesthetics.. Goodman and Gilman’s The Pharmacologic Basis of Therapeutics (ninth edition).

[52] Cannon SC, Robinson DA (1987). Loss of the neural integrator of the oculomotor system from brain stem lesions in monkey.. J Neurophysiol.

[53] Godaux E, Cheron G, Mettens P (1990). Ketamine induces failure of the oculomotor neural integrator in the cat.. Neurosci Lett.

