# Supplementary figures and images for: The Optokinetic Reflex as a Tool for Quantitative Analyses of Nervous System Function in Mice: Application to Genetic and Drug-Induced Variation
Source: PLoS One. 2008 Apr 30;3(4):e2055. doi: 10.1371/journal.pone.0002055 (PMC2323102; doi:10.1371/journal.pone.0002055)

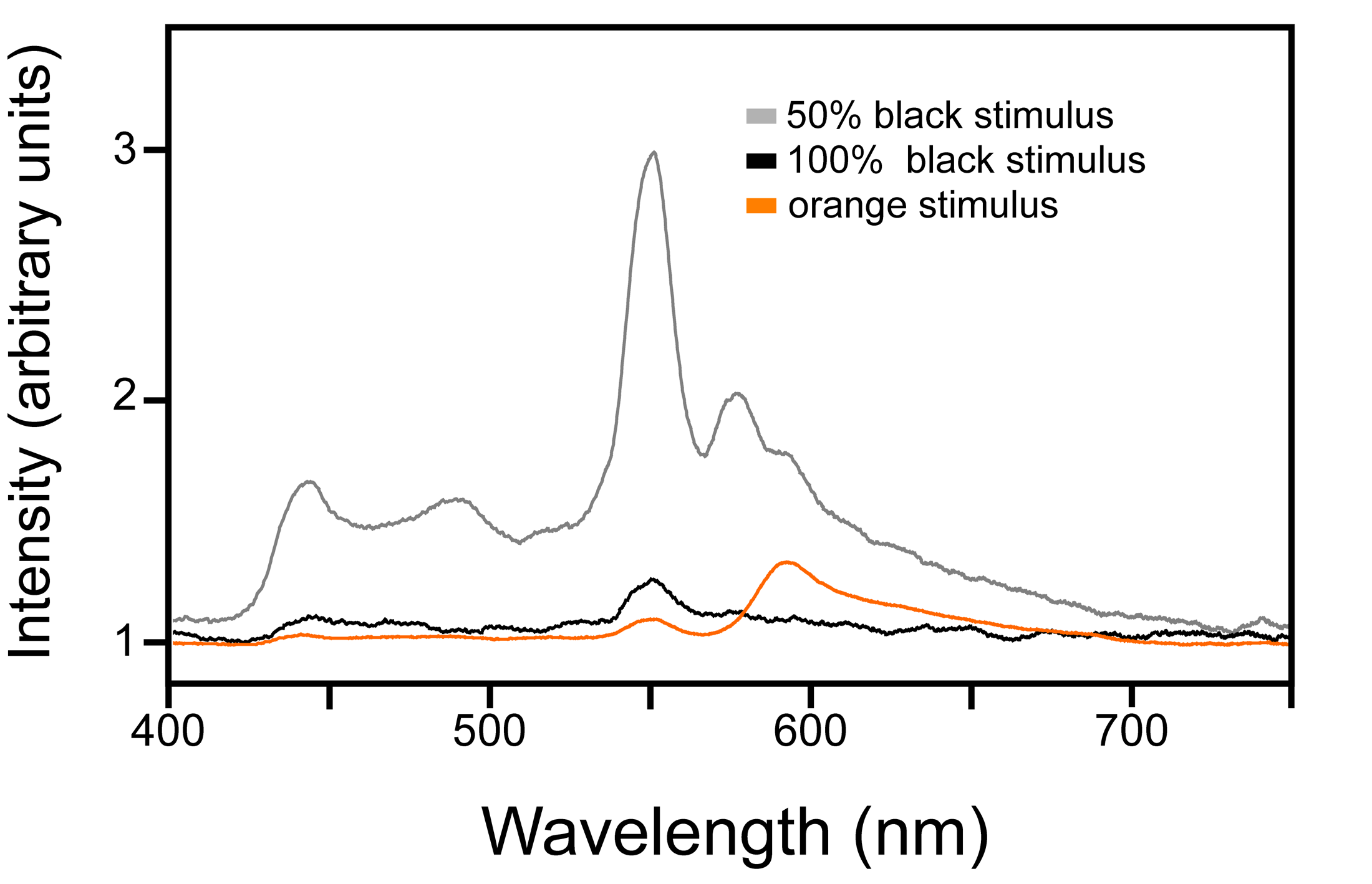

Supplement: Figure S1 — Spectral composition and relative intensities of OKR stimuli used for chromatic vs. gray scale experiments. Spectroradiometer measurements were made of the light reflected from the inner wall of the testing cylinder at the position of the mouse holder, when the LCD projector illuminated the cylinder with the indicated stimulus lights. (9.39 MB TIF) [file pone.0002055.s001.tif]

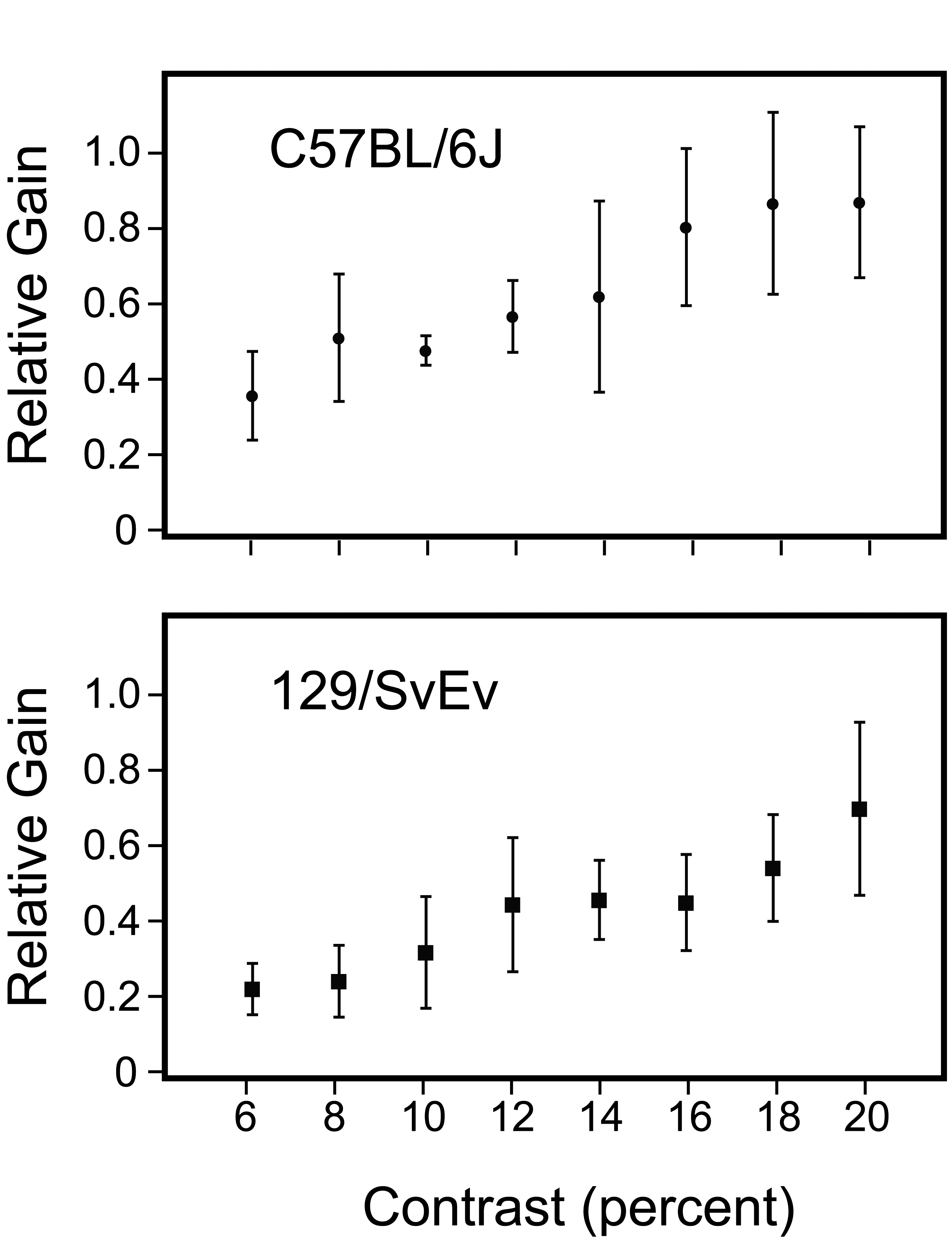

Supplement: Figure S2 — Relative gain in the slow component of the OKR as a function of percent contrast between the black and white stripes. Data are from the experiment shown in Figure 5A. Eye rotation is calculated based on a simplified model that places both the pupil image and the corneal reflections at the surface of the globe and assumes rotation about the center of a spherical globe (see Results section). A gain of 1.0 corresponds to an eye rotation that exactly tracks the stimulus. (9.20 MB TIF) [file pone.0002055.s002.tif]
